# Infinitesimals from a Scientific Standpoint

**Published:** 1896-02

**Authors:** J. M. Selfridge

**Affiliations:** Oakland, Cal.


### INFINITESIMALS FROM A SCIENTIFIC STANDPOINT.

J. M. Selfridge, M. D., Oakland, Cal.

Great interest has been manifested in the study of minute organisms ever since the microscope was first discovered, and from time to time, as improvements have been made in the magnifying powers of the instrument, new discoveries have been announced. About two decades ago great impetus was given in this direction by the discovery of those micro-fossils, the diatoms. Their study and classification, although pursued more for amusement than for the advancement of science, was productive of good in this—it stimulated the makers of optical instruments to still more improve the magnifying powers of the microscope, until at the present time it may with truth be said we have reached, for practical work, the out-limit of the power to magnify. With this improvement in optical instruments scientific investigation was greatly stimulated, and as a result great advancement in biological and histological knowledge has been made. One of the greatest triumphs of the modern microscope was the discovery and classification of those micro-organisms known as bacteria, some of them being so minute as to measure less than the one-seventy-thousandth of an inch in diameter. To the human intellect this is inconceivably small, and yet, in some instances, these minute objects are armed with ciliary projections which are so small that, after being magnified one hundred and sixty thousand times, they are scarcely visible. These micro-organisms are minute cells that contain protoplasm, "a particularly complex chemical substance out of which all living things, animals, and plants are formed." It is "made up of many atoms of carbon, hydrogen, oxygen, and nitrogen, with a small number of atoms of Sulphur and Phosphorus, more than a thousand of them in one molecule."

Small though these micrococci are, how infinitely minute must be the atoms of which they are composed when it takes a thousand of them to make one of the molecules of which they themselves are constructed.

While there are seventy or more elements, and "it appears that the individual atoms of each element are precisely alike," it does not follow that all molecules are of the same size. A molecule is made up of atoms chemically combined, and their size varies according to the number of atoms they contain. For example, a molecule of water is made up of three atoms—two of hydrogen and one of oxygen—while "a molecule of alum contains about one hundred," and, according to Mulder, a molecule of albumen contains nearly a thousand atoms; and, according to the same authority, the diameter of a molecule of alum would be equal to the one-ten-million-seven-hundred-and seventy-sixth thousandth of an inch, while the diameter of a molecule of albumen would be the one-five-millionth of *an inch.*

The molecules of matter are exceedingly interesting objects for study, but they can only be studied in combination, for when set free they develop a tremendous amount of energy, and the rapidity of their motions precludes the possibility of a *single* one being seen. For example, "a free molecule of hydrogen has a velocity of motion at ordinary temperatures of upwards of a mile in a second, and its direction of motion is changed millions of times in a second. There is every reason to believe that the molecules of all bodies are so perfectly transparent that they can no more be seen than the air, even if there were no difficulty from their smallness and their motions. If the atoms of a single element like hydrogen are so minute, so restless, and so transparent that no one can hope to see them so as to make out their forms, and what gives them their characteristic properties, what shall be said of the case of seventy or more elements similarly minute and restless and transparent, yet each one easily identified in several ways, physical and chemical?"

An atom is the chemist's unit, but "the term is not *now* understood to signify what is implied in its derivation, as something that cannot be divided, only as something that has not yet been broken up into smaller fragments."

Now, as Dolbear says, "Let it be granted that atoms are in the neighborhood of the one-fifty-millionth of an inch in diameter, then if a thousand of them are organized into a molecule, its diameter would be about the five-millionth of an inch." This being so, "a speck of protoplasm one-ten-thousandth of an inch in diameter would require not less than five hundred such molecules in a row to span it; and there would be no less than one hundred and twenty-five millions of such molecules in the small mass."

As I have already said, some of the micro-organisms are less than the one-seventy-thousandth of an inch in diameter, and yet they eat (by absorption), digest and excrete material substances so minute that the human mind grows dizzy at the thought of attempting to determine their dimensions. Minute though these objects are, they grow and multiply. This is done by what is called fission, or the cutting of themselves in halves or quarters. Now, for the sake of comparison, let us suppose that these minute objects are human beings. If this were so, a million of them could waltz on the point of the finest cambric needle. So far as science has been able to determine, these micro-organisms are the smallest of living beings, but, small as they are, they bear *no comparison* in point of minuteness to the *infinitesimal particles into which matter can be divided.* But, before entering upon the study of the divisibility of matter, it will be of interest to inquire into what is meant by matter. Several attempts have been made to define it, but it is more difficult to give a brief definition than one might at first imagine. "Whatever occupies space or whatever affects our senses" have been given as definitions, but, if we say that it is whatever occupies space—there may be any number of things in illimitable space that are not subject to any of the physical laws of which we have any knowledge. If we say whatever affects our senses, we are again going beyond our warrant, for electricity is capable of affecting several of our senses—sight, taste, feeling—and yet there is no good reason for thinking electricity to be matter. The best definition I have yet seen is given by Professor Dolbear, in his work on *Matter, Ether and Motion.* "*Whatever possesses the property of gravitative attraction*" is matter. From this definition it follows that the principles which Sir Isaac Newton and others applied to the large masses of matter apply with equal force to the smallest atom. Our best microscopes have enabled us to see particles of matter the one-hundred-thousandth of an inch in diameter, and yet this inconceivably minute particle is governed by the same laws as those that govern the earth, with a diameter of eight thousand miles, or the sun with its diameter of eight hundred thousand miles.

Although the smallest visible thing seen with the microscope is the one-hundred-thousandth of an inch in diameter, yet "there is no reason for thinking that such a degree of fineness is any approach to the ultimate fineness of the parts into which it is possible to divide matter. For a long time philosophers have considered whether or not there could, in the nature of things, be an actual limit to the divisibility of matter, so that the smallest fragment could not be again divided into two or more parts by the application of *appropriate means,* thus making matter infinitely divisible." As examples of this, "gold may be hammered into leaves no more than the one-three-hundred thousandth of an inch thick. Platinum can be drawn out into a wire finer than a spider's web—a single grain may be drawn into a mile of wire. A spider's web is sometimes so delicate that an ounce of it would reach three thousand miles, or from New York to London. No one would think it likely that such a web would be made up of a single row of atoms like a string of beads, for it would not seem probable that such a string could have such a degree of cohesion as spider's webs are known to possess. A grain of musk will keep a room scented for many years, giving out its particles to the currents of air to be wafted presently out-of-doors, yet in all this time the musk seems to lose but a little in weight." Faraday estimated that the particles of gold in the ruby liquid, made by the action of Phosphorus on a solution of gold, formed only the five-hundred-thousandth part of the volume of the liquid—that is, that one-five-hundred thousandth of each drop was gold, and yet the particles reflected light when rays of the sun were thrown into the liquid with a lens. "The spectroscope will indicate the millionth of a grain by the gas flame, and the color of a drop of water is appreciably changed by the one-three-millionth of a grain of fuschine. Some substances, like the essential oils, sulphuretted hydrogen, and the odor of flowers, can be perceived when the quantity is certainly less than the fifty-millionth of a grain." Dr. Thomson obtained sensibly appreciable quantities of sulphuret of lead, which, according to his computations, must have been divided into at least five hundred billion parts.

Professor Dolbear says there are five hundred millions of millions of millions of molecules in a cubic inch of gas. Again, "one-eighth of a grain of indigo dissolved in Sulphuric-acid will give distinctly blue color when dissolved in two and one-half gallons of water." Now, suppose the amount of water to be doubled, the blue color would doubtless disappear, but the particles of indigo would not be obliterated—they would be merely divided in halves, and this process of dilution might be carried on *ad infinitum*, and still particles of indigo would be present, for it is a well-established principle in science "that whatever else may decay, atoms do not, but remain as types of permanency through all imaginable changes." Think for a moment, as Dolbear says, of the wonderful "amount of intelligence associated with the minute brain structure of some of the smallest forms of animal life—say the ant, and, so far as such intelligence is associated with atomic and molecular brain structure, the size of the brain in the smallest ant, though measured in thousandths of an inch, is sufficiently large to involve billions of atoms, and the permutations possible are almost unlimited." But the most striking example of the extent to which matter may be divided and still manifest its presence, *by the exhibition of energy*, is given by Tyndall, who proved that a quantity of watery vapor, so small as to be absolutely inappreciable by any other test, increased the absorptive power of dry air to the obscure rays of heat to such an extent as to cause a marked difference in the deflection of the needle of a galvanometer.

It is difficult to understand how such minute particles of matter can affect the senses in any appreciable way, and yet we have something akin to it in the acute sense of smell of the dog. It is well known that he can track his master hours after the tracks have been made, showing very conclusively that minute particles of matter from the master's feet must have passed, not only through the leather of his boots, but have left characteristic matter at each foot-fall.

Ever since the discovery of cell structure it has been held that the primal cell *per se* was the seat of activity in all organized bodies. While this in a sense is true, we must not lose sight of the fact that the primal seat of life (and, therefore, of all physiological activities) is in that highly complex, that wonderful substance—protoplasm—that is contained *within* the cell, which, structureless though it be, is the wheel within a wheel whence emanates the power to build.

If we dissect this structureless mass, we will find it composed of numberless molecules, each molecule composed of from three to one thousand atoms, so curiously combined that they are the very seat of life. Sifted to its ultimates, the first physical form of all material bodies is the atom. Here, then, we have the order of growth. Atoms variously combined form molecules, and molecules, with their contained life-principle, constitute protoplasm, which, when in normal condition, is capable of organizing itself into cells, tissues, and organs.

"It was formerly thought that the cell was the unit of the physiologist; but, as the microscope was improved, and anatomical research continued, it became evident that the cell, with its more or less complicated structure, was itself built by the structureless protoplasm, which, as we have already seen, is composed of different kinds of atoms. Strange though it may seem, this structureless protoplasm is capable or organizing itself into cells and tissues in the same sense as atoms organize themselves into molecules and molecules into crystals of various sorts, having properties that depend upon the different kind of atoms, their number and arrangement in the molecules." Thus we see that atoms play an important part in the structure of the universe. To accomplish what is claimed for them, it is evident that they possess the property of chemism, and by some it is thought there is good reason for believing they are magnets. But, notwithstanding they possess these properties, conditions sometimes exist when they will not arrange themselves according to the laws of chemism or magnetism, a condition that resembles the living organism when disease prevents its normal activities. For example, "some supersaturated solutions seem unable to initiate the process of crystallization, but the smallest crystal of the substance starts it, and the whole body is solidified in a few seconds. Here it is evident that the crystal, taken as a nucleus, had a *field* that compelled other and similar molecular groups to arrange themselves in similar order. When two tuning-forks, having the same pitch, are separated from each other a distance of several feet, and one of them be made to produce a sound, the other one will be made to sound likewise by the action of the sound waves upon it. The effect is called sympathetic vibration. Other forks having different rates of vibration will not be similarly affected, so the vibrations in the air select out the particular fork having the same rate as the one vibrating, and cause it to enter into a similar state of vibration. Raise the damper of the piano and sing a sound of any particular note, then listen. The same note will be heard prolonged by the piano. The particular string which can give that pitch of sound has been thrown into similar vibrations and continues to sound as it would if caused to in any other way. When a single key of a piano is struck there is produced a musical sound. There is a definite pitch that is maintained. Strike half a dozen adjacent keys at once, and the effect is what we call a noise, though each component by itself would give a pleasing sound. Nearly every body has its own musical pitch, but if a number of bodies with different unrelated pitches are listened to at once, the effect upon the ear is a discordant one and is called a noise."

So it appears with a magnet. Any magnetic bodies in its field become magnetized there—that is, they are brought into the same physical state as the body that incited the field.

"Such physical fields are capable of compelling bodies within them to assume the state of motion or similar position or both as the body that produced the field, provided the substance itself be constituted molecularly like the first. It is a kind of induction common throughout the whole domain of physics." So also in the animal economy, "growth consists in the formation of similar cells out of suitable molecular constituents in the neighborhood."

From all this it is evident that there is a law of similars in science as well as in medicine, and the examples given above (which have been gleaned from scientific works) suggest a scientific explanation as to the manner in which the most similar remedy produces a cure. From what we have seen, it is evident that every medicinal substance is capable of producing what is known in science as a *field*. This being true, the remedy that is similar to the atoms and molecules that compose the primal cell, which is the seat of the disease, creates a healthy field in the vicinity of the diseased molecules, and, like the similar crystal in the supersaturated solution (to which reference has already been made), a healthy movement is inaugurated from within outward, which continues until the whole economy is restored to health.

From what we have already seen, it is evident that when, from the interference of any cause, the atoms and molecules of the protoplasm which constitute the primal cell are distuned, as Hahnemann has it, a healthy *field* or condition can only be established by the action of a similar substance or medicine, the vibrations of whose molecules are similar to the vibrations of the molecules of the diseased cell. Like the tuning-fork in the example given above, the disordered molecules of the primal cell will vibrate in a normal manner only when acted upon by a remedy whose molecules have similar vibrations; or, as Hahnemann teaches us, by a remedy that is able to produce symptoms like the symptoms of the disease. This being true, it is evident that to give more than one remedy at a time in the same case is unscientific. As every drug is capable of producing a *field* peculiar to itself, it would certainly be unscientific to produce two or more fields in the vicinity of the diseased cells at one and the same time. It would be like the example of the piano given above—a discord would be the inevitable result. The single remedy, therefore, is the only scientific mode of prescribing for the sick.

Science also suggests a reason for making the infinitesimal dilutions used in homœopathic practice. Atoms, as we have already seen, are the ultimates of all physical forms, and, to reach them successfully when diseased, it is evident that atoms should be used, for it is a well-established principle of science that atoms combine with atoms for which they have an affinity.

The method of dividing and subdividing medicines which Hahnemann found from experience to be the best adapted to the cure of disease, and especially of all chronic diseases, is, therefore, in full accord with the principles of science. It matters not, then, whether we view Hahnemann's teachings from the standpoint of the physician or the physicist, his conclusions are incontrovertible, for science and Homœopathy rest on the same foundation.

It may be said that the dilutions of Hahnemann, and especially of those of his followers, who have carried potencies much higher than those of the master, exceed in minuteness anything that has been attained by science. Admitting this to be true, it does not violate the teachings of science, for, as we have already seen, the conclusion of philosophers, is that "by the application of *appropriate means* matter may be infinitely divisible."

There is no reason, therefore, for supposing that the curative principle of drugs is separated from material substances in making the highest potencies.*

---

* This is taught by Hahnemann in *The Organon* (see note to Sec. 280), where he says: "Let these ordinary practitioners ask mathematicians to demonstrate the truth that, although a substance be divided into every so many parts, *some portion* of this substance, however minute, must still constitute each one of these parts; that the most inconceivably minute fractional particle never ceases to be *something* of the original substance, and hence that it can never become nothing."

There are those in the homœopathic school who will challenge this statement, for it is well known that their claim is that dynamization not only separates the "vital force" of drugs from the material, with which it is combined, but also increases its curative power. While this *may* be true, it is only a theory for which, so far as I am informed, there *is no analogy in science.* In fact, the conclusion of "all students of biology of the present age is that vital force as *an entity* has no existence, and it is the opinion of *all* biologists at the present time that there are no special forces of any kind—that all physiological phenomena whatever can be accounted for without going beyond the bounds of physical and chemical science." As to the origin of *life*, science is agnostic.

It has been said that when chemists "shall be able to form the substance protoplasm, it will possess all the properties it is now known to have, including what is called its life." That this statement is incorrect it is only necessary to remark that chemists *have* formed "the substance protoplasm," but they *cannot make it act.* This shows most conclusively that *life* is that *mysterious principle* which pervades all organized bodies, and without whose dynamic influence all physiological processes cease. While this is undoubtedly true, it is also equally true that when the phenomena of things about us and the functions within us are carefully studied and well understood, it will be found that *motion plays an important part in everything that occurs.* For example, heat is the result of the vibratory motion of atoms and molecules, while "electricity is a phenomenon of *rotary* molecules." "Light is undulatory movements, or ether waves, the source of which is the vibratory motions of the atoms and molecules of the sun, which come to us at the rate of one hundred and eighty-six thousand miles per second." Science teaches us that "all phenomena involves the motions of matter."

A favorite expression of homœopaths, and especially of the high dilutionists, is that disease is disturbed *vital force ;* but, if the conclusions of scientists are worthy of consideration, a better definition would be that disease is *disturbed atomic and molecular*

6

*motion*, which, as we have already seen, can be most readily restored to its normal condition by the administration of the most similar remedy in atomic quantities.

In close connection with the theory that disease is disturbed *vital force* is that other theory to which reference has just been made, viz., that the vital force or spiritual essence of drugs can be separated from its material relations and attached to other substances, such as sugar of milk or alcohol. To sustain this theory it is argued that certain substances which, in the crude state, are inert, become active remedies when carried through several degrees of potentization. That this is true of such drugs as *Gold, Vegetable Carbon, Silicea, Platinum,* and others, cannot be successfully denied. But to explain this really wonderful result it is not necessary to introduce a mysterious theory which requires us to believe that the curative principle of drugs is a spiritual essence. The attempt to maintain such a theory is not only a stumbling-block in the way of many minds, but it is contrary to all scientific experience. As is well understood, this so-called spiritual development is attained by potentization. But what is potentization? It is nothing more mysterious than the divisibility of matter according to an arbitrary but very convenient rule. Is there any mystery about its action in the case of those substances which, in the crude state, are inert? Certainly not. It merely sets free their atoms and molecules, and, as we have already seen, gives them an opportunity for greater freedom of action. An example of this is found in metallic Mercury, which, in its crude state, is absolutely inert, but when vaporized by heat and its molecules set free it becomes a most *potent* and *rapid* poison. If we admit that the curative power of drugs is a spiritual force that can be detached from its material mother, can it be made more spiritual and its curative power increased by being still further potentized? Skinner, who is an authority on homœopathic science, claims that it is *not* potentization, but *dilution*.* Is it possible to dilute that which is char-

---

*I do not agree with Skinner. It is undoubtedly potentization or the divisibility of matter by which its molecules and atoms are set free.

acterized by the *absence* of the properties that distinctly belong to matter? This would seem to involve an absurdity. It is certainly not scientific.

These thoughts are not uttered with a desire to reflect upon Hahnemann, for I have the most exalted opinion of his genius; but the facts set forth in this paper show how far he was in advance of the men of his time. The explanations given by Hahnemann in regard to the action of medicines can be accounted for on the ground that science, in his time, was in its infancy, and as the *results* (which his keen observation and experience had wrought out) could *not* be explained by science as it was then understood, he very naturally attributed whatever he could *not* explain to those spiritual influences for which he had the most *profound reverence.*

